# Relating Photoperiod and Outdoor Temperature With Sleep Architecture in Patients With Neuropsychiatric Sleep Disorders

**DOI:** 10.1111/jpi.70030

**Published:** 2025-01-07

**Authors:** Katy Sarah Weihrich, Frederik Bes, Jan de Zeeuw, Martin Haberecht, Dieter Kunz

**Affiliations:** ^1^ Institute of Physiology, Sleep Research & Clinical Chronobiology, Charité–Universitätsmedizin Berlin, corporate member of Freie Universität Berlin Humboldt‐Universität zu Berlin, and Berlin Institute of Health Berlin Germany; ^2^ Clinic for Sleep & Chronomedicine, St. Hedwig‐Hospital Berlin Germany

**Keywords:** humans, photoperiod, seasons, sleep wake disorders, sleep, REM, sleep, slow‐wave, temperature

## Abstract

While artificial light in urban environments was previously thought to override seasonality in humans, recent studies have challenged this assumption. We aimed to explore the relationship between seasonally varying environmental factors and changes in sleep architecture in patients with neuropsychiatric sleep disorders by comparing two consecutive years. In 770 patients, three‐night polysomnography was performed at the Clinic for Sleep & Chronomedicine (St. Hedwig Hospital, Berlin, Germany) in 2018/2019. Sleep times were adjusted to patients' preferred schedules, patients slept in, and were unaware of day‐night indicators. Digital devices and clocks were not allowed. Days were spent outside the lab with work or naps not allowed. After exclusions (mostly due to psychotropic medication), analysis was conducted on the second PSG‐night in 377 patients (49.1 ± 16.8 year; 54% female). Sleep parameters were plotted as 90‐day moving‐averages (MvA) across date‐of‐record. Periodicity and seasonal windows in the MvA were identified by utilizing autocorrelations. Linear mixed‐effect models were applied to seasonal windows. Sleep parameters were correlated with same‐day photoperiod, temperature, and sunshine duration. The MvA of total sleep time (TST) and REM sleep began a 5‐month‐long decline shortly after the last occurrence of freezing 24‐h mean temperatures (correlation of TST between 2018 and 2019 at 2‐month lag: *rs*_361_ = 0.87, *p* < 0.001; maximum peak‐to‐nadir amplitude: *ΔTST* ~ 62 min, *ΔREM* ~ 24 min). The MvA nadirs of slow wave sleep (SWS) occurred approximately at the autumnal equinox (correlation between 2018 and 2019: *rs*_361_ = 0.83, *p* < 0.001). Post hoc testing following significant linear mixed‐effect model indicate that TST and REM sleep were longer around November till February than May till August (*ΔTST* = 36 min; *ΔREM* = 14 min), while SWS was 23 min longer around February till May than August till November. Proportional seasonal variation of SWS and of REM sleep as percentages of TST differed profoundly (SWS = 31.6%; REM = 8.4%). In patients with neuropsychiatric sleep disorders living in an urban environment, data collected in 2018 show similar patterns and magnitudes in seasonal variation of sleep architecture as the 2019 data. Interestingly, whereas SWS patterns were consistent between years with possible links to photoperiod, annual variations of TST and REM sleep seem to be related to times of outside freezing temperature. For generalization, the data need to be confirmed in a healthy population. No clinical trial was registered.

## 1 Introduction

The Earth's rotation creates the most predictable variation in nature that affects life: the daily light‐dark cycle. An annual variation is superimposed on the cycle by the combination of Earth's motion around the Sun and its tilted axis of rotation relative to the orbital plane. In many species, these changes throughout the year induce seasonal variations in behavior and physiology, such as hibernation, migration, and seasonal breeding [1]. While the mechanisms behind these variations are well understood in many organisms [2], they remain less so in humans [3, 4, 5, 6, 7].

In humans, the conservation of photoperiodic responsiveness, including sleep, has been well documented [8]. Light exposure has a profound impact on human physiology, and in the prolonged absence of regular light cues, the sleep‐wake cycle will free‐run [9]. However, under strict light‐dark cycle protocols, the sleep‐wake cycle re‐establishes entrainment [10]. The degree of entrainment is influenced by light exposure factors such as intensity, spectral distribution, timing, and duration [8, 11, 12, 13, 14]. Overall, light is considered the strongest *zeitgeber* for the circadian pacemaker under natural conditions [15]. Given that natural light exposure varies seasonally in the form of daily photoperiod length, it is reasonable to expect that human sleep also exhibits seasonal variations.

Previous research has identified seasonal changes in hormone secretion and behavior of humans [16, 17]. At the extreme end of responses, some individuals experience seasonal affective disorder, which can be alleviated by light therapy [18]. However, the comparison of sleep logs, melatonin, and other circadian biomarkers recorded during summer and winter in urban environments gave inconsistent results [4, 19]. The influence of natural light on the circadian pacemaker is thought to be weakened in humans due to “voluntary control” over artificial light sources [15, 20]. Specifically, while markers of the circadian pacemaker (melatonin, plasma cortisol, and plasma prolactin) are tightly bound to the scotoperiods in humans, there is some evidence that seasonal changes in these processes are suppressed when individuals live in an urban environment [19, 21, 22]. Nevertheless, seasonal variations in subjective sleep length have been repeatedly confirmed [3, 6, 23]. But, to the best of our knowledge and with one exception [24], no significant seasonal variations in objective electrophysiologically recorded sleep length have been found.

Recent polysomnography (PSG) data from our group, collected in an urban environment, showed substantial variations in sleep architecture over the year in a large group of patients with neuropsychiatric sleep disorders who visited our sleep laboratory in 2019 [25]. We aimed to strengthen this previously reported evidence for seasonal variations, by extending the data set by 1 year. Furthermore, we aimed to explore links between the patterns in sleep architecture and environmental factors such as outdoor temperature, sunshine duration, and photoperiod by comparison of the 2 years.

## 2 Methods

The primary focus of PSG recording in the neuropsychiatric sleep laboratory of the Clinic for Sleep & Chronomedicine at the St. Hedwig Hospital in Berlin (52° N, 13° E, Germany), is to evaluate the representative sleep of patients without social constrains, including habitual sleep ability, timing, and duration. Extended descriptions of procedures can be found in the supplementary. The following features are of note: 1. to our knowledge, this sleep laboratory is unique in allowing patients to sleep at their preferred times and to sleep in; 2. the availability of three consecutive nights allows us to disregard the adaptation night, which is known to generate unrepresentative sleep (“first‐night‐effect” [26]); 3. the rooms are light‐ and sound‐proofed; 4. digital devices, monitors and alarm clocks are not allowed; as a result, patients are unaware of day‐night indicators and clock time. During daytime hours between lab‐nights patients are not allowed to stay in the hospital or to attend work. Naps are strictly forbidden.

**Figure 1:**
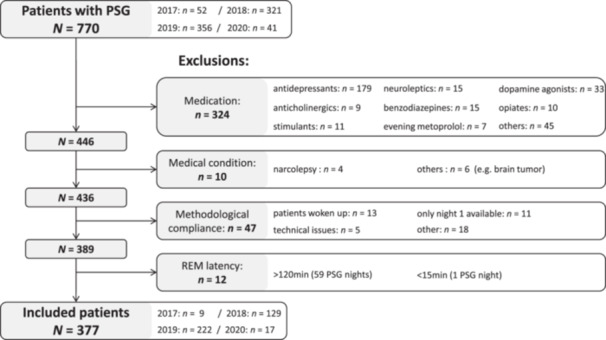
Patient flow chart depicting exclusion criteria. PSG = polysomnography.

The data set used in this study consisted of PSG data recorded between 17 November 2017 and 14 February 2020. The results of the analysis of some of the data recorded in 2019 were previously reported [25]. The years 2019 and 2018 were chosen since they were the most recent years before the COVID‐19 pandemic. The corresponding data set was extended in the present study with PSG data from an additional 37 patients with insomnia disorder. These patients were previously excluded because they had undergone a slightly different sleep protocol, which restricted the time in bed to exactly 8 h and is referred to henceforth as the 8h‐TIB‐Protocol. Since these patients were prediagnosed with insomnia disorder, they did not come close to 8 h of sleep. During the specified timeframe, all patients were evaluated for inclusion in this retrospective study. Patients gave written informed consent allowing the use of their anonymized data for research and publication. In total, PSG recordings from 770 patients were assessed. Of these patients, 393 were excluded due to conditions possibly disrupting neurotypical sleep architecture. Exclusion criteria are presented in Figure 1, with justification given in the supplementary. The included 377 patients (53.8% female) had a mean (**M**) age of 49.1 years with a standard deviation (SD) of 16.8 years. Sleep‐related diagnoses for the included patients were obtained from the medical discharge letter and are reported with descriptive statistics and monthly distribution of patients' age and gender in the Supporting Information S1: Table S1. Most prevalent diagnoses were: insomnia disorder (*n* = 248), parasomnia including RBD (*n* = 101), and depressive disorder (*n* = 82).

PSG recordings were conducted using a Rembrandt system (Monet 24‐CPU hardware, TMS International, Enschede, the Netherlands; Rembrandt 7.5 software, Medcare Automation, Amsterdam, the Netherlands), and were visually scored following the guidelines of the American Academy for Sleep Medicine Version 2.5 [27]. The PSG recording began with “lights‐off” as soon as a patient indicated readiness to sleep and setup was complete. The recording ended at “lights‐on”, representing the point when the patient requested to get out of bed. Sleep period time (SPT) was defined as the duration between sleep onset (the first two consecutive minutes (min) of sleep stages starting with N2, N3, or REM) and the end‐of‐sleep (the last two consecutive min of sleep stages N1, N2, N3, or REM). Total sleep time (TST) encompassed the accumulated duration of sleep stages N1, N2, N3, and REM between lights‐off and lights‐on. The time between lights‐off and sleep onset was defined as sleep latency, while the time between sleep onset and the first REM episode was defined as REM latency. Slow wave sleep (SWS) was identified as N3. Sleep stages were analyzed both in total minutes (min) and as a percentage of TST (%).

Environmental parameters in this paper encompassed weather parameters (temperature and sunshine duration) and an astronomical parameter (photoperiod). Daily photoperiod information (measured in hours from sunrise to sunset) for Berlin was sourced with permission from timeanddate.de [28]. Weather data were extracted from the Climate Data Center (CDC) of the Deutscher Wetterdienst (German Meteorological Service, DWD) [29, 30]. The meteorological station “Berlin‐Tempelhof” was selected, and daily measures for mean temperature in °C and sunshine duration in hours were extracted. Sunshine duration was measured as hours of direct solar radiation at a specific location. During the preliminary explorative investigation of the data, other available weather parameters (daily minimum and maximum temperature, air pressure and precipitation) were also explored, but not analyzed.

All data analysis was performed using MATLAB (The Mathworks Inc. Natick, Massachusetts, USA). To examine the relationship between sleep parameters and environmental factors, Spearman's rho correlations were computed, correlating raw sleep parameter data with the daily average of temperature, sunlight duration, and photoperiod. The correlations were performed as 1‐sided, left‐tail tests.

Since PSGs were recorded at irregular intervals, time series analysis of the raw data was not feasible. Attempts to resample the data set were discarded due to the uneven distribution of the included PSG records and the sometimes large time breaks between PSG records. Therefore, to evaluate the seasonality of the individual sleep parameter, a variety of methods were applied and considered together to evaluate if the sleep parameter show *consistent*, *some* or *no* evidence for seasonal pattern within our data set.

First, the data was visualized by applying a moving‐average (MvA) with a 90‐day window and a 1‐day slide. The 90‐day window allows for averaging over season, without selecting hard start and end dates. To enable plotting the MvA of the “**Continuous‐2‐Year data set**” from 01 January 2018 to 31 December 2019, the surrounding 45 days at the end of 2017 and the beginning of 2020 were included. Furthermore, MvAs were applied to the years 2018 (the “**2018 data set**”) and 2019 (the “**2019 data set**”) in isolation. To enable accurate plotting of the corners, the November/December data was added before the January data, and the January/February data was appended to the December data of the same year. At last, a MvA was created for the “**Averaged‐2‐Year data set**”, where the data recorded on the same day‐of‐year in different years were treated as the same date‐of‐record, thereby creating an averaged MvA between years.

**Figure 2:**
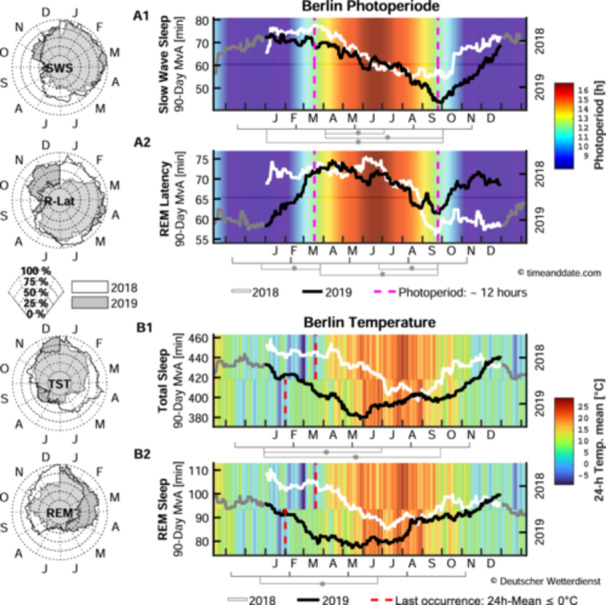
Seasonal variation of environmental factors and sleep architecture. Selected sleep parameters are given as 90‐day moving averages of the Continuous‐2‐Year data set (*N* = 377) for 2018 (white) and 2019 (black). Not‐depicted sleep parameters can be found in the Supporting Information S1: Figure S1. Plots are extended into the previous/next year (gray). Left: Spider plots illustrate each year's variation over time as a percentage of their minimum and maximum value. Right: Environmental parameters for 2018 (top) and 2019 (bottom) of (A) photoperiod in hours (timeanddate. com 28) and (B) daily mean temperature in °C (Deutscher Wetterdienst: CDC‐Portal 29). Red dashed lines: last instances when 24‐h mean temperature reached values below 0°C; Magenta dashed lines: days when the photoperiod is closest to 12h, at spring and autumnal equinox. Months are abbreviated at the first letter (i.e. J = January). * Significant Linear Mixed Model post hoc results between 91‐day‐long “seasonal windows” (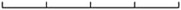) determined by the sleep parameters lag at minimum autocorrelation.

For visualizing, the Continuous‐2‐Year data set's MvA of the sleep parameters with environmental parameters as background was used (see Figure 2). The MvA was visually assessed for consistency of pattern between years and for occurrence of the acrophase (timing of the peak) and bathyphase (timing of nadir). To detect periodicity in the time series, autocorrelation was applied to the MvAs of the Continuous‐2‐Year data set, with lags iterated from 0 to 410 (1 lag = 1 day). Thus, the number of 410 lags covers exactly 50% of the data set (2 years, plus prior and subsequent 45 days). The number of lags at local correlation minimum (**MinLag**) was identified as an indicator for the time point at which the MvA pattern was most reversed. The number of lags at local correlation maximum (**MaxLag**) indicates the time point at which both years were most in phase with each other. MinLag closer to 26 weeks and MaxLag closer to 52 weeks provide some statistical evidence for existence and timing of the MvAs' seasonal pattern.

For statistical analysis of seasonal variation, “**seasonal windows**” were identified for the Averaged‐2‐Year, 2019, and 2018 datasets by separating the “year” into four not‐overlapping 91‐day long “seasonal” windows. The center of the “main” window was identified by calculating the MinLag for each data set and each sleep parameter. For example, MinLag = 25.86weeks would identify the center of the “main” seasonal window as Jun‐30 (i.e., 30 June 2018 and 30 June 2019) encompassing the time from May‐16 to Aug‐14, with the center of the “surrounding” seasonal windows being shifted by +91 days (Sep‐29: Aug‐15 till Nov‐13), ‐91 days (Mar‐31: Feb‐15 till May‐15) and +182 days (Dec‐30: Nov‐15 till Feb‐13). Analysis of variance for a linear mixed‐effects model (LMM) was performed with sleep parameters set as the dependent factors and seasonal windows as the fixed factor. Multiple comparison correction was done using the Bonferroni‐Holm method. Post hoc analysis, utilizing two‐sided independent t‐test between seasonal windows, was corrected using the false discovery rate [31].

To compare temporal consistency between years the 2018 and 2019 sections of the Continuous‐2‐Year MvA were correlated. Negative correlation was considered evidence against seasonality. Furthermore, rank‐correlation between the 2018 and 2019 sections were applied with 182 days backwards to 182 days forwards shifts. Shift with the highest positive correlation was identified as the “ideal” overlap between years. Only strong correlations are considered evidence for seasonality with variation between years.

## 3 Results

The year 2018 was exceptional because it had the highest overall average temperature and sunshine duration of the prior 30‐year period. In addition, it had an exceptionally long and cold winter followed by a swift transition to a long summer, with 24‐h mean freezing temperatures ending 2 months later in 2018 than 2019 (for elaboration see supplementary).

Linear mixed‐effect model of the Averaged‐2‐Year data set displayed a significant effect of seasonal windows for all evaluated sleep parameters, except for sleep latency (see Table 1, descriptive statistics reported in the Supporting Information S1: Table S3). Post hoc comparison (see Table 2) shows significantly longer durations for seasonal windows centered on December compared to June for TST, SPT, and REM sleep. SWS duration was significantly shorter for the seasonal windows centered in October compared to December and March. The timing and pattern of seasonal variation in SWS were similar when expressed as %TST or in minutes, and the same applies in this respect to the timing and pattern of REM sleep (see Supporting Information S1: Table S2, Figure S1 and Figure S2). The proportional seasonal difference of SWS (min) amounts to a 32% reduction (72 min around April vs. 49 min around October resulting in a 23 min difference), whereas the seasonal difference of REM sleep (min) amounts to a 14% reduction (see Supporting Information S1: Table S4). Longer REM latency post hoc results occurred centered on June and March compared to September. Additional analysis of the timing of sleep onset and end‐of‐sleep (see Supporting Information S1: Figure S4) showed no significant differences for sleep onset, despite pronounced variations in the 2018 (and slight variations in the 2019) MvA plots. However, end‐of‐sleep time displayed a significant effect of seasonal windows with earlier awakening around June than December.

**Table 1:** Linear mixed‐effect model results.

Sleep parameter		Averaged‐2‐year data set	2018 data set	2019 data set
Total Sleep	*F* (df), *p*	6.20 (3373), < 0.001*	1.29 (3125), = 0.282	2.95 (3218), = 0.034
Time [min]	MinLag [c.]	25.86 [Mar‐31, *Jun‐30*, *Sep‐29*, **Dec‐30**]	25.86 [Mar‐31, Jun‐30, Sep‐29, Dec‐30]	26.57 [Jan‐04, Apr‐05, Jul‐05, Oct‐04]
Sleep Period	*F* (df), *p*	3.80 (3373), = 0.010*	1.04 (3125), = 0.378	2.84 (3218), = 0.039
Time [min]	MinLag [c.]	25.57 [Mar‐29, *Jun‐28*, Sep‐27, **Dec‐28**]	18.86 [Feb‐11, May‐12, Aug‐11, Nov‐11]	25.43 [Mar‐28, Jun‐27, Sep‐26, Dec‐27]
Sleep Latency	*F* (df), *p*	0.25 (3373), = 0.858	1.04 (3125), = 0.378	0.96 (3218), = 0.412
[min]	MinLag [c.]	25.86 [Mar‐31, Jun‐30, Sep‐29, Dec‐30]	22.57 [Mar‐08, Jun‐07, Sep‐06, Dec‐07]	25.43 [Mar‐28, Jun‐27, Sep‐26, Dec‐27]
REM Latency	*F* (df), *p*	8.00 (3370), < 0.001*	2.19 (3,123), = 0.093	3.07 (3218), = 0.029*
[min]	MinLag [c.]	25.29 [**Mar‐27**, **Jun‐26**, *Sep‐25*, *Dec‐26*]	26.29 [Jan‐02, Apr‐03, Jul‐03, Oct‐02]	11.57 [**Mar‐22**, **Jun‐21**, *Sep‐21*, Dec‐22]
REM‐Sleep	*F* (df), *p*	5.04 (3373), = 0.002*	1.97 (3125), = 0.122	3.29 (3218), = 0.021*
[min]	MinLag [c.]	25.00 [Mar‐25, *Jun‐24*, Sep‐23, **Dec‐24**]	25.86 [Mar‐31, Jun‐30, Sep‐29, Dec‐30]	23.71 [*Mar‐16*, *Jun‐15*, Sep‐14, **Dec‐15**]
REM‐Sleep	*F* (df), *p*	3.79 (3373), = 0.011 *	0.70 (3125), = 0.551	3.06 (3218), = 0.029*
[% TST]	MinLag [c.]	24.86 [Mar‐24, *Jun‐23*, Sep‐22, **Dec‐23**]	18.29 [Feb‐07, May‐08, Aug‐07, Nov‐07]	25.57 [Mar‐29, Jun‐28, Sep‐27, Dec‐28]
Slow Wave	*F* (df), *p*	8.47 (3373), < 0.001*	4.15(3,125), = 0.008 *	4.95 (3218), = 0.002*
Sleep [min]	MinLag [c.]	26.43 [**Jan‐03**, **Apr‐04**, *Jul‐04*, *Oct‐03*]	25.43 [**Mar‐28**, *Jun‐27*, *Sep‐26*, **Dec‐27**]	26.43 [**Jan‐03**, **Apr‐04**, Jul‐04, *Oct‐03*]
Slow Wave	*F* (df), *p*	7.10 (3373), < 0.001*	2.64 (3125), = 0.052	5.69 (3218), < 0.001*
Sleep [% TST]	MinLag [c.]	26.29 [**Jan‐02**, **Apr‐03**, **Jul‐03**, *Oct‐02*]	25.57 [Mar‐29, Jun‐28, Sep‐27, Dec‐28]	26.29 [**Jan‐02**, **Apr‐03**, **Jul‐03**, *Oct‐02*]
	*N*	377 (n2017 = 52, n2020 = 41)	129	222

*Note:* LMM (sleep parameter ~ seasonal windows around MinLag) was conducted on four consecutive seasonal windows centered at the MinLag. bold * = significant after Bonferroni‐Holm correction applied to sleep parameter groups; bold = center of seasonal window with post hoc showing significant longer durations; *italic* = center of seasonal window with post hoc showing significant shorter durations.

Abbreviations: [c.], center of 91‐day seasonal windows with date format = “MMM‐dd” (e.g., “Jun‐30” is the center of the time from “May‐16” to “Aug‐14”); df, degrees of freedom; MinLag, lag at local minimum of autocorrelation (in weeks); MvA, 90‐day moving average.

**Table 2:** Significant post hoc results with large effect size for total sleep time, rem sleep and slow wave sleep in minutes.

Sleep parameter	Centre of seasonal windows and directionality	mean Δ	95% CI	Test statistic
**Averaged‐2‐Year data set**				
Total sleep time	Dec‐30 > Jun‐30	36.12 min	(35.55, 36.69)	*t* (213) = 3.95, *p* < 0.001, *d* = 0.54
	Dec‐30 > Sep‐29	33.53 min	(32.91, 34.16)	*t* (188) = 3.41, *p* < 0.001, *d* = 0.50
REM‐sleep	Dec‐24 > Jun‐24	14.43 min	(14.20, 14.66)	*t* (211) = 3.93, *p* < 0.001, *d* = 0.54
Slow wave sleep	Jan‐03 > Oct‐03	20.27 min	(19.97, 20.57)	*t* (186) = 4.14, *p* < 0.001, *d* = 0.62
	Apr‐04 > Oct‐03	23.48 min	(23.14, 23.81)	*t* (163) = 4.32, *p* < 0.001, *d* = 0.68
**2018 data set**				
Slow wave sleep	Mar‐28 > Jun‐27	18.46 min	(18.4, 18.87)	*t* (73) = 2.59, *p* = 0.012, *d* = 0.60
	Mar‐28 > Sep‐26	20.1 min	(19.53, 20.49)	*t* (64) = 2.54, *p* = 0.013, *d* = 0.63
**2019 data set**				
Slow wave sleep	Jan‐03 > Oct‐03	20.25 min	(19.85, 20.66)	*t* (109) = 3.07, *p* = 0.003, *d* = 0.59
	Apr‐04 > Oct‐03	24.60 min	(24.16, 25.4)	*t* (98) = 3.42, *p* < 0.001, *d* = 0.69

*Note:* Selected post hoc results for significant Linear Mixed‐effect Model (sleep parameter ~ seasonal windows around MinLag [lag at local minimum of autocorrelation], after Bonferroni‐Holm correction) and after False Discovery Rate corrections for multiple comparison. Only results with a large effect size are presented (*d* ≥ 0.5), the extended table can be found in the supplementary, Table S2. Center of 91‐day seasonal windows reported with date format = “MMM‐dd” (e.g., “Jun‐30” is the center of the time from “May‐16” to “Aug‐14”).

Abbreviations: 95% CI, 95% Confidence Intervals implementing bootstrap resampling (*n* = 1000); d, Cohens' d; df, degrees of freedom; Δ, difference between 91‐day seasonal windows.

The MvA plots of the Continuous‐2‐Year data set (see Figure 2B) for TST, SPT (see supplementary, Figure S1), and REM sleep show identifiable singular annual acrophases and bathyphases that occurred about 2‐months apart between years. In 2018, acrophase occurred around spring and bathyphase in summer, while 2019 shows a winter acrophase and spring bathyphase. Differences between peak and nadir reached around 62 min for TST (*MvAΔTST*_*09Jan18/25Aug18*_ = 61.1 min, *MvAΔTST*_*28May19/31Dec19*_ = 63.0 min), around 24 min for REM sleep (*MvAΔREM*_*09Jan18/10Jul18*_ = 25.2 min, *MvAΔREM*_*15May19/27Dec19*_ = 22.3 min), and around 27 min for SWS (*MvAΔSWS*_*17Mar18/05Oct18*_ = 23.5 min, and *MvAΔSWS*_*09Jan19/28Sep19*_ = 30.0 min). SWS's MvA (see Figure 2A1) is consistent between 2018 and 2019 with a spring acrophase and autumn bathyphase.

REM latency (see Figure 2A2) exhibited some consistency between 2018 and 2019, overlapping strongly from March to August, but displays opposing directionality between October and February. Sleep latency did not display any consistent pattern between years or pronounced annual acrophases and bathyphases (see supplementary, Figure S1).

Details on the Continuous‐2‐Year datasets MinLag, MaxLag, and the correlation between the 2018 and 2019 MvA are provided in Table 3. MaxLag hints towards an overall consistent annual pattern for REM latency and SWS parameters, while for TST, SPT, and REM sleep strongest similarity between years is indicated at around 9.5 months. For the latter parameters (TST, SPT, REM [min] and REM [%]), “ideal” overlap between 2018 and 2019 sections occurred at forward shifts between 1.9 and 3.1 months. Even without shift, SWS parameters reach similarly strong significant overlap between years. While the shift for REM latency to reach “ideal” overlap is the smallest for all parameters, the correlations are weak both without and after shift.

**Table 3:** Correlations used to evaluate similarity between the 2018 and 2019 90‐day moving‐average data.

Sleep parameter	MinLag/MaxLag	Correlation between years (without shift)	Shift towards ideal overlap ← →	Correlation between years (after shift)
	**in weeks**	**rs (df** = **361),** ***p***	**in days**	**rs (df** = **361),** ***p***
Total sleep time [min]	26.29/41.43	0.53, < 0.001	→ 69 →	0.87, < 0.001*
Sleep period time [min]	23.29/41.43	0.03, 0.546	→ 92 →	0.81, < 0.001*
Sleep latency [min]	NA/NA	−0.38, < 0.001	← 125 ←	0.30 (237), < 0.001
REM latency [min]	26.57/53.57	0.41, < 0.001	← 11 ←	0.46 (351), < 0.001
REM sleep [min]	23.29/42.86	0.39, < 0.001	→ 57 →	0.91, < 0.001*
REM sleep [%TST]	24.86/39.71	0.16, 0.002	→ 88 →	0.70, < 0.001
Slow wave sleep [min]	26.86/53.86	0.83, < 0.001*	← 29 ←	0.88 (333), < 0.001*
Slow wave sleep [%TST]	28.43/54.43	0.76, < 0.001	← 47 ←	0.88 (315), < 0.001*

*Note:* Notes on autocorrelation lags (MinLag and MaxLag): Autocorrelation conducted on the Continuous‐2‐Year data set (*N* = 377). MinLag and MaxLag were converted into weeks (7 daily lag = 1 weekly lag). NA applies when no MinLag/MaxLag were identified.

Notes on correlation between years: Rank correlation conducted on the Continuous‐2‐Year data set (*N* = 377) between the 2018 and 2019 MvA without shifting the 2019 data, and after applying shift to “ideal” overlap of the 2018 and 2019 MvA data at the reported shift; * Strong significant correlation (rs > 0.8).

Abbreviations: df, degrees of freedom (default df, if not otherwise specified); MinLag/MaxLag, lag at local minimum/maximum of autocorrelation; MvA, 90‐day moving average; NA, not available; rs, Spearman rank correlation; TST, total sleep time.

All significant Spearman's rho correlations between sleep parameters and environmental parameters show weak negative directionalities (see Table 4). TST, SPT, and REM sleep displayed a significant decrease in duration with increasing photoperiod length, sunshine duration, and average daily temperature, while SWS only showed significant correlation with temperature. While SWS did not decrease and increase along with the photoperiod, the start of the decrease in SWS (30 March 2018 and 13 March 2019) approximately coincided with the spring equinox, from which the daily photoperiod became longer than 12 h. Additionally, the start of the increase in SWS (05 October 2018 and 28 September 2019) approximately coincided with the autumnal equinox, when the photoperiod became shorter than 12 h (see Figure 2A1).

**Table 4:** Correlation between sleep parameters with environmental parameters.

Parameter		Photoperiod [h]	Sunshine duration [h]	Temperature [°C]
Total sleep time [min]	*rs* (*df* = 375), *p*	−0.21, < 0.001*	−0.14, 0.003*	−0.20, < 0.001*
Sleep period time [min]	*rs* (*df* = 375), *p*	−0.19, < 0.001*	−0.15, 0.001*	−0.18, < 0.001*
Sleep latency [min]	*rs* (*df* = 375), *p*	0.00, 0.507	0.02, 0.616	−0.02, 0.382
REM latency [min]	*rs* (*df* = 372), *p*	0.10, 0.972	0.11, 0.981	0.07, 0.924
REM‐sleep [min]	*rs* (*df* = 375), *p*	−0.21, < 0.001*	−0.12, 0.009*	−0.18, < 0.001*
REM‐sleep [% TST]	*rs* (*df* = 375), *p*	−0.14, 0.003*	−0.06, 0.141	−0.10, 0.031
Slow wave sleep [min]	*rs* (*df* = 375), *p*	−0.09, 0.050	−0.04, 0.205	−0.17, 0.001*
Slow wave sleep [% TST]	*rs* (*df* = 375), *p*	−0.01, 0.440	0.02, 0.636	−0.09, 0.034*

*Note:* Spearman rho rank correlation between sleep parameters (*N* = 377) and environmental parameters for the same day as date of record. Environmental parameters: photoperiod (timeanddate. com [1]), sunshine duration and mean temperature (Data: Deutscher Wetterdienst, CDC‐Portal [2, 3]). * significant after Bonferroni‐Holm correction applied to parameter groups.

Abbreviations: df, degrees of freedom; TST, total sleep time.

The meteorologically exceptional year 2018 shows an approximately 1.5‐month delay in high‐ and low‐temperature periods compared to 2019 (see Figure 2B). This delay closely mirrors the 2‐month shift between years observed in TST and REM sleep. In both years, peaks in TST and REM sleep were reached around the first occurrence, while decline began around the last occurrence of 24‐h mean freezing temperatures. For REM sleep, the decline onset was immediate, whereas for TST, it occurred with a 2‐to‐4‐week delay. Both sleep parameters started to increase again around the first occurrence of average daily temperature > 27°C.

## 4 Discussion

Presented sleep architecture data from our 2018 patients with neuropsychiatric sleep disorders living in an urban environment support the seasonal patterns of sleep architecture that we previously reported for a similar patient group in 2019 [25]. Once again, variations in sleep parameters were observed throughout the year, with fluctuations of approximately 60 min in TST, 25 min in REM sleep, and 30 min in SWS. SWS duration was proportionally 32% shorter in autumn than in spring, while REM sleep duration in minutes was decreased by around 14% in summer compared to winter. Differences in seasonal changes between SWS and REM sleep seem to be even more pronounced when given as proportion of TST (SWS∝ = 32% and REM∝ = 8%). Besides magnitude, patterns of variation in SWS, TST, and REM sleep were consistent between years. Timing of SWS variations around the spring and autumn equinox was almost identical. In contrast, annual variations of TST and REM sleep occurred in parallel but almost 2 months apart in 2018 versus 2019. Most interesting this 5‐months decline of TST and REM sleep started around 2 weeks after average 24‐h outside temperature rose above freezing.

All of our observations and analyses hint towards the existence of a seasonal variation of SWS, with bathyphase occurring almost exactly around the autumn equinox and acrophase around the spring equinox in both years. Similar timing of seasonal variation in SWS were also found in previous research employing monthly recordings under climate‐controlled laboratory settings [32]. The consistency of the SWS pattern, despite strong variation in weather parameters between years, may point to the conclusion that SWS is more associated with photoperiod than with other environmental parameters. A substantial phase difference between the 90‐day MvA of SWS and photoperiod exists (depicted in the supplementary, Figure S3), exemplified by the nadir of SWS, occurring around the autumn equinox on 23 September, and the nadir of the photoperiod, occurring at the winter solstice (21 December). Thus, the lack of a significant correlation between the patients' SWS duration and same‐day photoperiod length in Table 4 can be mainly attributed to this approximately 90° phase difference. Furthermore, the negative correlation between the patients’ SWS duration and the time course of outdoor temperature can be understood with a similar line of reasoning. The time course of outdoor temperature lags roughly 45° behind that of the photoperiod (see supplementary, Figure S5). Thus, the phase difference of the patients' SWS duration with the outdoor temperature is even larger than with the photoperiod, approximately 135°, tending towards phase opposition and thus resulting in a negative correlation in Table 4.

Also of note in our data is the overlap between amount of SWS and REM latency decline around the same time period (see Figure 2A2). Interdependency between SWS and REM sleep pressure (e.g., with napping) was shown to influence REM latency [33], while reduced SWS pressure was shown to occur with reduced REM latency [34]. REM sleep itself is known to be under circadian control [35], which in turn is modulated by light via melatonin secretion [15]. Melatonin offset was delayed in winter compared to summer under natural light conditions during camping, but not under electrical light conditions at home [19]. Nevertheless, there is some evidence for earlier melatonin peak amplitude in summer compared to winter [36], and lower melatonin peak amplitude in combination with higher ambient light exposure in summer [37]. Furthermore, studies looking at sleep architecture between seasons tend to find longer REM sleep duration for winter than summer periods [38, 39]. While in‐lab recordings of “in‐bed” and “wake‐up” time have been previously found to occur earlier in summer, shorter TST durations in summer were not significant [36, 38]. A recently conducted in‐depth review indicated that sleep onset and end‐of‐sleep tends to occur later in summer than in winter [40]. Our data for 2018 that shows about 20 min earlier sleep onset and end‐of‐sleep time at the start of June compared to December (see supplementary Figure S4) seems to corroborate this finding.

For TST and REM sleep, our analysis mostly hints towards seasonal variation [40] but with an around 2‐month shift between years. Bathyphases occurred earlier than the summer solstice in 2019 and later than the summer solstice in 2018, resulting in the bathyphases of the Averaged‐2‐Year MvA to coincide with the summer solstice, and in significant correlations between these parameters and photoperiod. However, 2018 was a special year with the longest period of mean 24‐h temperature below freezing for the last 30 years and a long warm summer. The patterns of variation of TST and REM sleep between years were impressively similar. In both years, decreasing time courses were in parallel, with a 2‐month difference over a 5‐month period, and started around 2 weeks after the last occurrence of outdoor 24‐h mean freezing temperatures. Thus, the variation in TST and REM sleep between years might be related to annual climate variability.

Somewhat surprisingly, the “season” aspect is rarely reported on in studies utilizing the gold standard PSG, neither in patient studies concerning sleep nor in experimental human studies that have effects of light or circadian‐driven parameters as key outcomes [41]. Only few studies recorded sleep architecture parameters, such as SWS, over various seasons. And even less report descriptive statistics across time‐of‐record, or statistical analysis of seasonal variations [25, 38, 39, 42, 43, 44, 45, 46, 47]. Most of these studies reported cohorts of rather small populations and children, or were conducted under extreme photoperiod duration in Antarctica. In contrast, the present data set represents a large patient cohort that was intensively screened for factors disturbing the patient's normative sleep (such as psychotropic medications, “organic” induced sleep disturbances, being woken up, or first‐night‐effect), resulting in an exclusion rate of 51%.

### 4.1 Limitations

Although, the Averaged‐2‐Year data set shows significant differences between seasonal windows, TST was not significant for the 2018 and 2019 datasets, which may be explained by an insufficient cohort size in the individual years. Statistical testing of seasonal data, even with clear visual seasonal trends, remains difficult when handling small cohort sizes [48]. The overall variability between patients (e.g., SD_*TST*_ = 69.5 min) is higher than the maximum variability between monthly means (e.g., *M*_*TST*_ *Δ*_*Jun/Dec*_ = 52.4 min). A required minimum cohort size of 372 patients was estimated for TST and 276 patients for SWS (see supplementary, Table S5). Whereas this threshold was reached for the Averaged‐2‐Year data set (*N* = 377), 222 patients were included in 2019, and only 129 patients were included in 2018, which thus might explain the lack of significant results for the 2018 data set. It can be concluded, that a very large data set is required to test for seasonal trends in sleep architecture when analyzing PSG data using a between‐subject design.

Another limitation of our study is the heterogeneity of the patients' diagnoses and the high prevalence of co‐morbidities, such as depression and insomnia disorder. Shorter TST in 2019 may be attributed to the high number of patients with insomnia disorder. Of the largest diagnostic group, patients with insomnia disorder, only 73 patients were included in 2018 and 152 in 2019 (see supplementary, Table S1). Of course, it would be of interest to examine the extent of seasonal variation within the different diagnostic groups. Again, the groups were too small for analysis. Including further years could enable analysis of individual diagnostic groups if averaged across years; however, this exceeded the scope of the current paper.

Last but not least, we did not measure circadian timing, such as dim light melatonin onset, or assessed individual light exposure patients were subjected to before the laboratory visit. Nevertheless and corroborated by our data, it is well known that humans seem to adapt to lighting conditions over longer terms of weeks and maybe even months [18, 49, 50].

## 5 Conclusion

The present study provides compelling evidence of substantial seasonal variation in sleep architecture in humans, even when living in an urban environment and among individuals with neuropsychiatric sleep disturbances. Since sleep deprivation, such as in our patients with insomnia disorder, diminishes circadian drives of sleep‐wake patterns [51], it is reasonable to speculate that seasonal variation in sleep may be even more pronounced in healthy individuals. Our data suggest that different environmental factors may play distinct roles in driving seasonal variation in sleep parameters. Duration of SWS appears to be critically associated with photoperiod, whereas duration of TST and REM sleep seems closely linked to outdoor temperature. If the present findings can be replicated in a healthy adult population, it will become imperative to consider such factors when establishing normative data for sleep architecture or examining the functions of sleep.

Discussions about the role of light in guiding the timing of the circadian *zeitgeber* are still going strong. Recently, the importance of considering reduced sensitivity to light in summer was put into focus when reporting light exposure studies [41]. Conversely, the influence of seasonal elements (such as solar noon) on self‐reported sleep is said to be outshined by clock time and individually recorded light exposure [52].

In conclusion, even though artificial lighting allows sleep, work, and school schedules to be uncoupled from natural light‐dark cycles, the impact of natural light on human physiology and behavior should not be underestimated – even in an urban environment; to be confirmed in a healthy population.

## Author Contributions

Contribution of all authors throughout the duration of the study was ensured by regular meetings. Dieter Kunz procured resources and coordinated responsibilities. Jan de Zeeuw, Frederik Bes, and Dieter Kunz planned study concept and design. Katy Weihrich and Martin Haberecht screened patients for exclusion criteria, with supervision of final patient inclusion by Dieter Kunz. Frederik Bes and Martin Haberecht supervised sleep stage scoring done by the clinics' medical technical staff. Martin Haberecht developed a novel automated and anonymizable PSG data extraction. Katy Weihrich, Frederik Bes, and Jan de Zeeuw designed the analysis plan, executed by Katy Weihrich. Katy Weihrich extracted environmental parameters. Katy Weihrich drafted the manuscript and prepared figures a nd tables. Frederik Bes and Dieter Kunz critically edited the manuscript. All authors edited and reviewed the manuscript, and all authors approved the final version. The authors declare no conflict of interest.

## Ethics Statement

Ethical review and approval were not required for the study on human participants in accordance with the local legislation and institutional requirements. Exemption confirmation by Ethical Board ‐ Charite (EA4/197/24).

## Consent

Patients gave written informed consent allowing the use of their anonymized data for research and publication.

## Conflicts of Interest

The authors declare no conflicts of interest.

## Supporting information

Supporting information.

## Data Availability

The data that support the findings of this study are available from the corresponding author upon reasonable request.
