# Supplementary material for: Relating Photoperiod and Outdoor Temperature With Sleep Architecture in Patients With Neuropsychiatric Sleep Disorders
Source: J Pineal Res. 2025 Jan 7;77(1):e70030. doi: 10.1111/jpi.70030 (PMC11707406; doi:10.1111/jpi.70030)
Supplement: Supplementary file 1 — Supporting information. [file JPI-77-e70030-s001.docx]

**Supplementary** **Material**

for

Relating Photoperiod and Outdoor Temperature with Sleep Architecture in Patients with Neuropsychiatric Sleep Disorders

Katy Sarah Weihrich,^1,2^ Frederik Bes,^1,2^ Jan de Zeeuw,^1,2^ Martin Haberecht,^2^ Dieter Kunz^1,2*^

^1^Institute of Physiology, Sleep Research & Clinical Chronobiology, Charité–Universitätsmedizin Berlin, corporate member of Freie Universität Berlin, Humboldt-Universität zu Berlin, and Berlin Institute of Health, Berlin, Germany

^2^Clinic for Sleep & Chronomedicine, St. Hedwig Hospital, Berlin, Germany

^*^Corresponding author: Dieter Kunz; Klinik für Schlaf- & Chronomedizin, St. Hedwig-Krankenhaus, Große Hamburger Straße 5-11, 10115 Berlin, Germany; dieter.kunz@charite.de

## Exclusion criteria

Typically, patients spent 3 consecutive nights in our clinic (see supplementary, *4.2. Polysomnography recording procedure*) and the second night was used for evaluation. In cases where methodological or technical issues arose, or if the REM latency threshold was breached on the second night, the data from the third night were considered for inclusion. If both the second and third nights’ data were excluded, the exclusion criterion for the second night was reported.

- 1. *Exclusion medication*

As part of our routine protocol, patients were asked to pause any medications that could influence sleep for at least 2 weeks prior to undergoing polysomnography (PSG), when feasible. Patients that took medication that affects sleep within 5-times the half-life of the medications were excluded from the study. To identify the potential use of drugs or substances known to disturb sleep, especially rapid eye movement (REM) sleep, patients’ medical histories, physicians’ instructions for the PSG, and patients’ self-reported medication intake on the day of PSG were carefully reviewed. Furthermore, a psychiatric sleep expert was consulted to determine acceptable time frames between the last intake of an exclusion medication and the date of PSG.

- 1. *Medical condition*

Patients with medical conditions known to affect sleep architecture, such as narcolepsy, were excluded from the study.

- 1. *REM Latency*

Extreme REM latency values can indicate skipped REM sleep ^1^, or undisclosed use or recent discontinuation of REM-suppressing medications or substances ^2^. Additionally, short REM latencies during PSG (REM latency < 15 minutes) are a part of the diagnostic criteria for narcolepsy according to the Fifth Edition of the Diagnostic and Statistical Manual of Mental Disorders (DSM-5) ^3^. As a result, REM latencies outside of the described thresholds were excluded from the analysis in the present study.

- 1. *Methodological compliance*

As described below (see supplementary, *4.2. Polysomnography recording procedure*), some patients had to be woken up for medical or diagnostic procedures or due to the 8h-TIB-Protocol. Patients who were still sleeping during “lights-on” or displayed major inconsistencies between the second and third night were excluded.

# Patient diagnosis

Patients attend our outpatient clinic at their own request or referred by their physician. After evaluation of our standard 17-pages questionnaire including six psychometric tests (PSQI: Pittsburgh Sleep Quality Index ^4^, ESS: Epworth Sleepiness Scale ^5^, FSS: Fatigue Severity Scale ^6^, MCTQ: Munich ChronoType Questionnaire ^7^, D-MEQ: German translation of the Morningness-Eveningness-Questionnaires ^8,9^, and SPAQ: Seasonal Pattern Assessment Questionnaire ^10^) patients are interviewed by a neuropsychiatric sleep specialist. PSG is performed when indicated according to standard guidelines except patients with sleep related breathing disorder. Thus primarily included are patients with movement in sleep that may indicate a parasomnia (e.g. REM sleep behavior disorder), circadian rhythm disorder such as due to shiftwork or because of insomnia disorder. Patients with insomnia disorder and/or depression are first treated ambulatory, most often with cognitive behavioral therapy (CBT-I) or with medication. In cases where there is suspicion of sleep apnea, patients are screened using ambulatory polygraphy and if positive referred to treatment by a pneumologist. Patients with insomnia disorder are only referred to the PSG if outpatient screening such as actigraphy and therapy are considered insufficient pointing to a possible organic component.

In total 177 of the 377 included patients (47%) were diagnosed with insomnia disorder without an organic sleep disturbance. The exclusion of patients that took psychotropic medication (e.g. antidepressants, see **Figure 1**) excluded a substantial amount of patients with depression.

Seasonal environmental changes can affect sleep disorders and co-morbidities, like higher nighttime temperatures in summer which increase insomnia disorder prevalence, which may be exacerbated by climate change in the future ^11^. And the seasonal variation in light exposure has a well known effect on seasonal affective disorder (SAD), with SAD patients sleeping longer during the winter and showing sleep architecture changes like less delta sleep and higher REM density, which can be reversed by bright light therapy ^12^. In patients with non-seasonal depression no such clear variation over the year was found ^12^. Yet, in our cohort that included a large group of insomnia disorder patients but very few SAD patients we do also find longer sleep duration in winter. Whether this longer sleep during winter differs from the general healthy population remains to be investigated by future research in large healthy cohorts.

1. **Patients distribution throughout the year**

| There was an uneven distribution of included PSG records across time. During the study period, 10.3% of all weeks included no patients, 32.5% of weeks included 1 to 2 patients, 40.2% of weeks included 3 to 5 patients, and 17.1% of weeks included 6 to 10 patients. Due to factors such as the holiday season, the longest break between records during the study was 23 days, with 3 breaks lasting more than 2 weeks (all between December-16 and January-08).  **Table S1. Patients’ demographics in number of patients by month and category** | | | | | | | | | | | | | | | | | |
| --- | --- | --- | --- | --- | --- | --- | --- | --- | --- | --- | --- | --- | --- | --- | --- | --- | --- |
| DoR | Total | Gender | | Age Group | | Age | Diagnosis | | | | | | | | | TIB  = 8h | alt.  N. |
| Group | *N* | ♂ | ♀ | < 60 | >= 60 | M±SD | Ism | Para | Dpr | srBD | PLM | RBD | RLS | Hyp | None |  |  |
| 17-11 | 4 | 2 | 2 | 2 | 2 | 63.7±14.6 | 3 | - | 2 | 1 | - | 1 | - | - | - | - | - |
| 17-12 | 5 | 3 | 2 | 5 | - | 35.8± 6.0 | 4 | 1 | 3 | - | - | - | - | - | - | - | 1 |
| 18-01 | 6 | 4 | 2 | 6 | - | 44.4± 6.8 | 3 | 2 | 2 | - | - | 1 | - | - | - | - | 1 |
| 18-02 | 18 | 6 | 12 | 16 | 2 | 41.7±14.9 | 10 | 5 | 2 | 4 | 3 | - | - | 1 | 1 | - | 3 |
| 18-03 | 14 | 9 | 5 | 12 | 2 | 46.6±17.0 | 7 | 1 | 1 | 8 | 1 | 2 | - | - | - | - | 5 |
| 18-04 | 8 | 2 | 6 | 7 | 1 | 44.7±12.3 | 8 | 1 | 4 | - | - | - | - | - | - | - | 1 |
| 18-05 | 11 | 7 | 4 | 8 | 3 | 46.2±21.6 | 3 | 6 | 2 | 2 | - | 4 | - | 1 | - | - | - |
| 18-06 | 17 | 12 | 5 | 9 | 8 | 55.0±16.9 | 8 | 1 | 2 | 3 | - | 7 | - | - | - | - | 3 |
| 18-07 | 10 | 4 | 6 | 5 | 5 | 57.1±16.5 | 6 | 4 | 1 | 3 | 2 | 2 | - | - | - | 2 | 4 |
| 18-08 | 14 | 7 | 7 | 9 | 5 | 54.8±16.2 | 9 | 1 | 2 | 1 | - | 2 | 2 | - | 2 | 2 | 4 |
| 18-09 | 7 | 1 | 6 | 5 | 2 | 45.1±18.2 | 2 | 3 | 1 | 1 | - | 1 | 1 | 2 | - | - | - |
| 18-10 | 8 | 5 | 3 | 6 | 2 | 47.6±19.2 | 4 | 2 | 2 | 2 | 1 | - | - | 1 | 1 | 1 | 2 |
| 18-11 | 5 | 2 | 3 | 4 | 1 | 52.6±14.2 | 5 | - | - | - | 2 | - | 1 | - | - | 1 | 2 |
| 18-12 | 11 | 5 | 6 | 7 | 4 | 50.3±16.1 | 8 | 2 | 1 | 2 | - | - | - | 2 | - | 4 | 3 |
| 19-01 | 21 | 9 | 12 | 13 | 8 | 51.4±17.0 | 8 | 7 | 4 | 5 | 5 | 5 | 1 | 2 | 1 | 1 | - |
| 19-02 | 18 | 10 | 8 | 12 | 6 | 49.7±15.4 | 11 | 2 | 2 | 4 | 3 | 1 | 1 | 3 | 1 | 5 | 3 |
| 19-03 | 16 | 6 | 10 | 10 | 6 | 51.3±17.3 | 11 | 6 | 4 | 4 | 3 | 2 | 2 | - | - | 3 | 1 |
| 19-04 | 17 | 5 | 12 | 12 | 5 | 50.4±15.5 | 9 | 8 | 5 | 6 | 2 | 1 | 2 | 2 | - | 3 | 2 |
| 19-05 | 18 | 8 | 10 | 14 | 4 | 46.5±17.8 | 13 | 4 | 8 | 2 | 2 | 1 | 1 | 1 | - | 6 | 4 |
| 19-06 | 22 | 11 | 11 | 14 | 8 | 51.9±16.3 | 14 | 8 | 7 | 6 | 2 | 3 | 3 | 1 | - | 4 | 4 |
| 19-07 | 21 | 12 | 9 | 14 | 7 | 48.6±17.9 | 16 | 5 | 4 | 6 | 6 | 3 | 2 | 1 | - | 2 | 2 |
| 19-08 | 14 | 5 | 9 | 9 | 5 | 48.8±16.6 | 11 | 4 | 5 | 3 | 2 | - | 2 | - | - | 2 | 2 |
| 19-09 | 17 | 7 | 10 | 11 | 6 | 51.4±18.2 | 15 | 3 | 5 | 2 | 3 | - | 3 | - | - | 5 | 3 |
| 19-10 | 21 | 11 | 10 | 11 | 10 | 57.0±15.2 | 17 | 9 | 3 | 5 | 2 | 1 | 1 | - | - | 6 | 4 |
| 19-11 | 18 | 5 | 13 | 15 | 3 | 44.9±12.0 | 15 | 10 | 4 | 6 | - | 1 | 1 | 1 | - | - | 4 |
| 19-12 | 19 | 8 | 11 | 18 | 1 | 46.5±14.4 | 12 | 7 | 4 | 3 | 4 | 3 | 2 | 1 | 1 | - | 2 |
| 20-01 | 10 | 6 | 4 | 10 | - | 39.1±14.1 | 7 | 4 | 2 | 1 | 1 | - | 2 | 1 | - | - | 5 |
| 20-02 | 7 | 2 | 5 | 7 | - | 39.5±13.0 | 7 | 5 | - | 1 | - | - | 1 | - | - | - | 2 |
| 2018 | 129 | 64 | 65 | 94 | 35 | 49.0±17.3 | 73 | 28 | 20 | 26 | 9 | 19 | 4 | 7 | 4 | 10 | 28 |
| 2019 | 222 | 97 | 125 | 153 | 69 | 50.0±16.5 | 152 | 73 | 55 | 52 | 34 | 21 | 21 | 12 | 3 | 37 | 31 |
| Total | 377 | 174 | 203 | 271 | 106 | 49.1±16.8 | 246 | 111 | 82 | 81 | 44 | 41 | 28 | 20 | 7 | 47 | 67 |
| **Abbreviations:** alt. N. = alternative Night used; DoR = date of record; Dpr = depression; Hyp = hypersomnia; Ism = insomnia; M±SD = mean±standard deviation; None = The suspected diagnoses were not confirmed and no other sleep-related disorders were diagnosed; Para = other parasomnias; srBD = sleep-related breathing disorders; RLS = restless-leg syndrome; PLM = periodic limb movement; RBD = REM-sleep behavior disorder; ♂ = male; ♀ = female. **Note:** Data grouped by individual month (dateformat = yy-mm, e.g. 18-01 = January 2018). Data reported as number per group unless specified. | | | | | | | | | | | | | | | | | |

## Sleep laboratory procedures

- 1. *Procedure history*

The Clinic for Sleep- & Chronomedicine at St. Hedwig-Hospital in Berlin, Germany, was founded in 2008, with diagnosis and treatment initiation in neurologic/psychiatric sleep disorders as the major aim. Patients are referred to or initiate contact themselves to our outpatient clinic. Diagnosis is performed in clinical interview (including a battery of psychometric testing), sometimes supported by several weeks of actigraphy. Depending on indication – referring to criteria of the International Classification of Sleep Disorders (ICSD-3; AASM, 2014) – patients may undergo diagnostic video-supported polysomnography (vPSG). After final diagnosis, treatments are initiated or proposed and patients return to their referring physician.

- 1. *Polysomnography recording procedure*

The patients are instructed to come to the sleep laboratory between 7 pm and 8 pm. The actual time depends on the patients’ prefered bed time. They are allowed to bring personal items (such as a specific pillow) and leave them in their room during the stay. Electrodes are attached between 7 and 9 pm and subsequently, patients are requested to stay in their room, but not to sleep, until the start of the PSG. The patients’ habitual sleep times during weekends and holidays serve as a baseline for scheduling the recordings. The night guard tells the patients to enter the bed around 10 minutes prior to appointed lights-out time. Alarm clocks are not permitted, although some patients need to be awakened in preparation for medical or diagnostic procedures, such as the Multiple Sleep Latency Test (MSLT). Each bedroom is equipped with heaters, and patients can adjust them to their comfort. Moreover, to ensure minimal noise and light exposure, triple windows with complete black-out shutters and soundproof doors are installed.

During the usual procedure (the “standard protocol”), patients undergo PSG measurements for 3 consecutive nights. The sleep laboratory can accommodate up to 6 patients simultaneously, with sleep recorded in individual single-bedrooms. Recordings are conducted either from Monday night until Thursday morning or from Friday night until Monday morning. For the sake of obtaining “most representative” sleep, patients on the days of the 3-night hospital stay are not allowed to stay in their rooms, but asked to spend time in the city. Patients are not allowed to work. Daytime naps are strictly forbidden.

The first nights in the laboratory served as an adaptation to the artificial laboratory situation and were excluded from data selection. Second nights were used for diagnosis and were the main focus of the analysis in this study. Third nights were typically used for evaluating treatment initiation, such as light exposure or administering medication. As such, third nights were considered as alternative nights for inclusion in the analysis if the second night was spoiled, by e.g. technical errors, and if no exclusion criteria applied for the night. Data from alternative nights were used for 67 patients (see supplementary, Table S1). To extend the dataset, additionally PSG recordings were included that had not been previously considered for analysis. To meet the criteria of the 8h-TIB-Protocol between July 2018 and October 2019, patients with insomnia disorder had to have spent exactly 8 hours in bed. The protocol remained otherwise unchanged from the standard protocol. Additional methodological exclusion criteria were applied for these patients: Recordings where patients were still sleeping during “lights-on” were excluded, assuming that they have not yet achieved their typical sleep need. Moreover, consecutive PSG recordings were evaluated by a physician to assess whether the patients had fulfilled their typical sleep need for the night before the point of “lights-on”.

- 1. *Data extraction*

For the previous data collection ^13^, sleep parameters were extracted in 2020 from automatically generated laboratory reports. To ensure consistency between the datasets, sleep parameters for the present dataset were extracted directly from the Rembrandt system’s output files using an internally developed sleep parameter extraction algorithm.

- 1. *Notes on data analysis*

# Since PSGs were recorded at irregular intervals, time series analysis of the raw data was not feasible. Attempts to resample the data were discarded due to an uneven distribution of, and intermittent time breaks between included PSG records.

## Results Supplementary

| **Table S2. Significant Post hoc results for the Linear Mixed-effect Model** | | | | |
| --- | --- | --- | --- | --- |
| Sleep Parameter | centre of seasonal windows & directionality | mean / *median* Δ | 95% CI | Test Statistic |
| **Averaged-2-Year dataset** | | | | |
| Total Sleep Time [min] | Dec-30 > Jun-30 | 36.12 min | (35.55, 36.69) | t(213)=3.95, p<.001, d=0.54 * |
|  | Dec-30 > Sep-29 | 33.53 min | (32.91, 34.16) | t(188)=3.41, p<.001, d=0.50 * |
| Sleep Period Time [min] | Dec-28 > Jun-28 | 30.4 min | (29.42, 30.65) | t(213)=3.03, p=.003, d=0.41 |
| REM Latency  [min] | Jun-26 > Sep-25 | 12.39 min | (12.20, 12.58) | t(171)=4.07, p<.001, d=0.62 * |
|  | Mar-27 > Sep-25 | 13.70 min | (13.51, 13.88) | t(157)=4.42, p<.001, d=0.70 * |
|  | Mar-27 > Dec-26 | 8.9 min | (7.89, 8.28) | t(199)=2.79, p=.006, d=0.40 |
| REM-Sleep [min] | Dec-24 > Jun-24 | 14.43 min | (14.20, 14.66) | t(211)=3.93, p<.001, d=0.54 * |
| REM-Sleep  [% of TST] | Dec-23 > Jun-23 | 1.95 % | (1.90, 1.99) | t(209)=2.85, p=.005, d=0.39 |
| Slow Wave Sleep [min] | Jan-03 > Oct-03 | 20.27 min | (19.97, 20.57) | t(186)=4.14, p<.001, d=0.62 * |
|  | Apr-04 > Oct-03 | 23.48 min | (23.14, 23.81) | t(163)=4.32, p<.001, d=0.68 * |
|  | Apr-04 > Jul-04 | 12.40 min | (12.11, 12.69) | t(187)=2.51, p=.013, d=0.37 |
| Slow Wave Sleep | Jan-02 > Oct-02 | 4.5 % | (3.97, 4.12) | t(187)=3.47, p<.001, d=0.51 * |
| [% of TST] | Apr-03 > Oct-02 | 5.51 % | (5.43, 5.58) | t(163)=4.36, p<.001, d=0.68 * |
|  | Jul-03 > Oct-02 | 3.17 % | (3.10, 3.25) | t(173)=2.61, p=.010, d=0.40 |
| **2018 dataset** | | | | |
| Slow Wave Sleep [min] | Mar-28 > Jun-27 | 18.46 min | (18.4, 18.87) | t(73)=2.59, p=.012, d=0.60 * |
|  | Mar-28 > Sep-26 | 20.1 min | (19.53, 20.49) | t(64)=2.54, p=.013, d=0.63 * |
|  | Dec-27 > Sep-26 | *26.78 min* | (26.12, 27.44) | U=203.50(26,28), p=.006, rb=-0.38 |
| **2019 dataset** | | | | |
| REM Latency  [min] | Mar-22 > Sep-21 | 10.32 min | (10.7, 10.58) | t(93)=2.66, p=.009, d=0.55 * |
|  | Jun-21 > Sep-21 | 9.77 min | (9.55, 9.100) | t(113)=2.67, p=.009, d=0.50 |
| REM-Sleep  [min] | Dec-15 > Jun-15 | *11.11 min* | (10.86, 11.35) | U=1237.50(59,61), p=.003, rb=-0.27 |
|  | Dec-15 > Mar-16 | *12.12 min* | (11.78, 12.46) | U=1043.00(59,51), p=.006, rb=-0.26 |
| Slow Wave Sleep [min] | Jan-03 > Oct-03 | 20.25 min | (19.85, 20.66) | t(109)=3.07, p=.003, d=0.59 * |
|  | Apr-04 > Oct-03 | 24.60 min | (24.16, 25.4) | t(98)=3.42, p<.001, d=0.69 * |
| Slow Wave Sleep [% of TST] | Jan-02 > Oct-02 | 4.82 % | (4.72, 4.92) | t(109)=2.91, p=.004, d=0.56 * |
|  | Apr-03 > Oct-02 | 6.69 % | (6.58, 6.79) | t(97)=3.87, p<.001, d=0.78 * |
|  | Jul-03 > Oct-02 | 4.61 % | (4.52, 4.71) | t(108)=3.01, p=.003, d=0.58 * |
| **Abbreviations:** 95%CI = 95% Confidence Intervals implementing bootstrap resampling (n=1000); d = Cohens' d; df = degrees of freedom; min = minute; rb = rank-biserial correlation; TST = total sleep time; Δ = difference between 91-day seasonal windows (date format = MMM-dd). N**otes:** Post hoc results only reported for significant Linear Mixed-effect Model (sleep parameter ~ seasonal windows around MinLag [lag at local minimum of autocorrelation], after Bonferroni-Holm correction) and after False Discovery Rate corrections for multiple comparison. * strong (rb ≥ 0.8) or large (d ≥ 0.5) effect size. | | | | |

| **Table S3. Descriptive Statistics for the Linear Mixed-effect Model (Format = Date Range ¶ N: M±SD; Median)** | | | | | |
| --- | --- | --- | --- | --- | --- |
| Sleep Para-meter | Dataset | seasonal window  - 91 days | seasonal window at Min Lag | seasonal window   + 91 days | seasonal window  + 182 days |
| Total Sleep Time [min] | 2-Year | 15.Feb - 15.May | 16.May - 14.Aug | 15.Aug - 13.Nov | 15.Nov - 13.Feb |
|  |  | 89: 418.0±68.5; 424.5 | 98: 402.1±72.7; 408.5 | 73: 405.0±71.4; 412.0 | 117: 438.4±61.8; 442.0 |
|  | 2018 | 15.Feb - 15.May | 16.May - 14.Aug | 15.Aug - 13.Nov | 15.Nov - 13.Feb |
|  |  | 39: 439.3±53.9; 438.5 | 37: 419.1±66.8; 432.0 | 26: 417.0±84.8; 430.0 | 27: 441.2±50.3; 435.5 |
|  | 2019 | 20.Feb - 20.May | 21.May - 19.Aug | 20.Aug - 18.Nov | 20.Nov - 18.Feb |
|  |  | 51: 398.7±76.2; 406.0 | 61: 394.7±73.0; 404.0 | 49: 398.7±61.9; 400.5 | 61: 427.6±64.3; 434.5 |
| Sleep Period Time [min] | 2-Year | 13.Feb - 13.May | 14.May - 12.Aug | 13.Aug - 11.Nov | 13.Nov - 11.Feb |
|  |  | 88: 464.8±63.7; 464.5 | 98: 450.6±74.3; 455.8 | 74: 455.9±68.4; 450.8 | 117: 480.6±71.0; 480.0 |
|  | 2018 | 28.Dec - 27.Mar | 28.Mar - 26.Jun | 27.Jun - 25.Sep | 27.Sep - 26.Dec |
|  |  | 38: 479.3±59.1; 470.5 | 35: 479.4±65.5; 482.0 | 32: 455.5±72.0; 461.5 | 24: 472.5±61.5; 472.5 |
|  | 2019 | 12.Feb - 12.May | 13.May - 11.Aug | 12.Aug - 10.Nov | 12.Nov - 10.Feb |
|  |  | 50: 453.7±62.7; 459.8 | 60: 436.6±76.0; 441.2 | 47: 453.5±60.0; 441.0 | 65: 472.8±75.9; 481.5 |
| Sleep Latency [min] | 2-Year | 15.Feb - 15.May | 16.May - 14.Aug | 15.Aug - 13.Nov | 15.Nov - 13.Feb |
|  |  | 89: 25.5±23.1; 17.5 | 98: 23.5±16.0; 20.0 | 73: 25.7±20.5; 20.0 | 117: 25.4±19.9; 20.0 |
|  | 2018 | 23.Jan - 22.Apr | 23.Apr - 22.Jul | 23.Jul - 21.Oct | 23.Oct - 21.Jan |
|  |  | 40: 22.8±23.6; 16.5 | 38: 23.2±14.9; 20.2 | 30: 28.9±21.6; 25.2 | 21: 19.8±15.4; 14.0 |
|  | 2019 | 12.Feb - 12.May | 13.May - 11.Aug | 12.Aug - 10.Nov | 12.Nov - 10.Feb |
|  |  | 50: 27.6±21.2; 20.0 | 60: 23.2±17.6; 18.0 | 47: 23.1±18.2; 19.0 | 65: 27.9±23.0; 20.5 |
| REM Latency [min] | 2-Year | 11.Feb - 11.May | 12.May - 10.Aug | 11.Aug - 09.Nov | 11.Nov - 09.Feb |
|  |  | 86: 73.5±22.0; 70.8 | 99: 72.0±22.1; 70.0 | 74: 59.8±17.2; 58.2 | 115: 65.2±20.1; 65.5 |
|  | 2018 | 18.Feb - 18.May | 19.May - 17.Aug | 18.Aug - 16.Nov | 18.Nov - 16.Feb |
|  |  | 38: 72.9±22.2; 65.5 | 38: 71.7±24.3; 67.2 | 27: 60.3±16.9; 61.0 | 24: 66.9±21.3; 66.0 |
|  | 2019 | 07.Nov - 05.Feb | 06.Feb - 06.May | 07.May - 05.Aug | 07.Aug - 05.Nov |
|  |  | 61: 65.1±22.3; 62.5 | 46: 72.3±21.6; 70.8 | 66: 71.4±22.0; 72.5 | 49: 61.9±16.1; 62.0 |
| REM-Sleep [min] | 2-Year | 09.Feb - 09.May | 10.May - 08.Aug | 09.Aug - 07.Nov | 09.Nov - 07.Feb |
|  |  | 88: 89.7±27.0; 89.8 | 101: 83.8±26.7; 82.5 | 76: 92.1±30.1; 89.8 | 112: 98.2±27.0; 95.8 |
|  | 2018 | 15.Feb - 15.May | 16.May - 14.Aug | 15.Aug - 13.Nov | 15.Nov - 13.Feb |
|  |  | 39: 100.8±24.0; 94.0 | 37: 87.3±23.5; 89.0 | 26: 94.8±32.9; 96.8 | 27: 99.9±27.8; 106.0 |
|  | 2019 | 31.Jan - 30.Apr | 01.May - 30.Jul | 31.Jul - 29.Oct | 31.Oct - 29.Jan |
|  |  | 51: 81.3±27.2; 81.0 | 61: 81.7±26.4; 82.0 | 51: 89.1±30.1; 81.0 | 59: 95.3±28.9; 92.5 |
| REM-Sleep  [% TST] | 2-Year | 08.Feb - 08.May | 09.May - 07.Aug | 08.Aug - 06.Nov | 08.Nov - 06.Feb |
|  |  | 91: 21.0±4.5; 21.0 | 101: 20.6±4.8; 20.3 | 75: 22.4±5.5; 22.1 | 110: 22.5±5.1; 22.9 |
|  | 2018 | 24.Dec - 23.Mar | 24.Mar - 22.Jun | 23.Jun - 21.Sep | 23.Sep - 22.Dec |
|  |  | 35: 23.0±4.4; 22.8 | 36: 21.6±4.0; 21.0 | 33: 22.3±5.4; 23.2 | 25: 21.5±5.4; 21.6 |
|  | 2019 | 13.Feb - 13.May | 14.May - 12.Aug | 13.Aug - 11.Nov | 13.Nov - 11.Feb |
|  |  | 50: 20.2±4.9; 19.9 | 61: 20.1±4.7; 19.9 | 46: 22.3±5.7; 22.0 | 65: 22.2±5.5; 21.9 |
| Slow Wave Sleep [min] | 2-Year | 19.Feb - 19.May | 20.May - 18.Aug | 19.Aug - 17.Nov | 19.Nov - 17.Feb |
|  |  | 89: 72.3±35.5; 74.5 | 100: 60.0±31.9; 56.8 | 76: 49.0±33.4; 52.0 | 112: 69.4±33.0; 72.0 |
|  | 2018 | 12.Feb - 12.May | 13.May - 11.Aug | 12.Aug - 10.Nov | 12.Nov - 10.Feb |
|  |  | 38: 76.7±31.5; 80.8 | 37: 58.2±30.2; 55.5 | 28: 56.9±31.0; 54.8 | 26: 75.3±28.0; 87.2 |
|  | 2019 | 19.Feb - 19.May | 20.May - 18.Aug | 19.Aug - 17.Nov | 19.Nov - 17.Feb |
|  |  | 50: 70.0±37.8; 71.2 | 61: 61.0±32.8; 57.0 | 50: 45.2±34.5; 36.8 | 61: 65.4±34.4; 61.0 |
| Slow Wave Sleep  [% TST] | 2-Year | 18.Feb - 18.May | 19.May - 17.Aug | 18.Aug - 16.Nov | 18.Nov - 16.Feb |
|  |  | 89: 17.4±8.1; 17.5 | 99: 15.0±7.8; 14.2 | 76: 11.9±7.9; 11.8 | 113: 15.9±7.8; 16.2 |
|  | 2018 | 13.Feb - 13.May | 14.May - 12.Aug | 13.Aug - 11.Nov | 13.Nov - 11.Feb |
|  |  | 38: 17.6±7.6; 17.8 | 37: 14.1±7.4; 13.5 | 28: 13.6±7.5; 15.3 | 26: 17.2±6.5; 18.4 |
|  | 2019 | 18.Feb - 18.May | 19.May - 17.Aug | 18.Aug - 16.Nov | 18.Nov - 16.Feb |
|  |  | 50: 17.5±8.5; 17.6 | 61: 15.6±7.8; 14.3 | 49: 11.0±8.2; 9.2 | 62: 15.7±8.6; 14.4 |
| **Abbreviations:** M = mean; N = number of patients within the seasonal window; SD = standard deviation; 2-Year = Averaged-2-Year dataset; 2018 = 2018 dataset; 2019 = 2019 dataset. **Notes:** LMM was conducted on four consecutive seasonal windows centered at the MinLag (lag at local minimum of autocorrelation). Reported are decriptive statistics at the identified seasonal windows for each sleep parameter for each dataset. Format = "date range (date format = dd.MMM) [linebreak] N: mean±standart deviation; median". | | | | | |

**Figure S1. Seasonal variation of environmental factors and sleep architecture**. Non-selected sleep parameters are given as 90-day moving averages of the Continuous-2-Year dataset (*N* = 377) for **2018** (white) and **2019** (black). Plots are extended into the previous/next year (gray). Left: Spider plots illustrate each year’s variation over time as a percentage of their minimum and maximum value. Right: Environmental parameters for 2018 (top) and 2019 (bottom) of photoperiod in hours (timeanddate.com ^14^) or daily mean temperature in °C (Deutscher Wetterdienst: CDC-Portal ^15^). Months are abbreviated at the first letter (i.e. J = January). * Significant Linear Mixed Model post hoc results between 91-day-long “seasonal windows” (
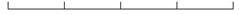
) determined by the sleep parameters lag at minimum autocorrelation.

| **Table S4. Proportional seasonal variation of selected significant post hoc results.** | | | | | | |
| --- | --- | --- | --- | --- | --- | --- |
| **Sleep  Parameter** | **Center of Seasonal Window** | | **Mean** | | **Seasonal Difference**  **Δ** | **Proportional Seasonal Variation ∝** |
|  | seasonal maximum | seasonal minimum | seasonal maximum | seasonal minimum |  |  |
| **TST (min)** | Dec-30 | Jun-30 | 438.4 min | 402.1 min | 36.3 min | 8.3 % |
| **SWS (min)** | Apr-04 | Oct-03 | 72.3 min | 49.0 min | 23.3 min | 32.2 % |
| **SWS (% TST)** | Apr-03 | Oct-02 | 17.4 % | 11.9 % | 5.5 % | 31.6 % |
| **REM (min)** | Dec-24 | Jun-24 | 98.2 min | 83.8 min | 14.4 min | 14.7 % |
| **REM (% TST)** | Dec-23 | Jun-23 | 22.5 % | 20.6 % | 1.9 % | 8.4 % |
| **Abbreviations:** REM = REM sleep; SWS = Slow wave sleep; TST = total sleep time;  **Notes**: Post hoc results selected from **Table S2**.  Seasonal Difference (Δ) = seasonal maximum – seasonal minimum.  Proportional Seasonal Variation (∝) = (seasonal difference (Δ) / seasonal maximum) x 100. | | | | | | |

The 90-day moving average of sleep latency does not provide evidence for seasonal variation. The general shape of seasonal variation for sleep period time (SPT) is similar to that of TST. The pattern of REM sleep expressed as % of TST (**%**) is similar compared to REM sleep expressed in minutes (**min**), and the same applies to SWS as % of TST compared to SWS in minutes. Therefore, the variations in sleep stages do not appear to be a mere result of changes in TST (see **Figure S2**). Decreases in SWS (%) coincide with rapidly increasing REM (%) in August (see **Figure S2.A**). The SWS (%) nadir also coincides with a peak in N2 (%) (see **Figure S2.B**). The SWS (%) increases in autumn and winter occur at the expense of N2 (%) (see **Figure S2.C**). But increases in N2 (%) in spring appear to occur at the expense of REM (%) (see **Figure S2.D**).

Figure S2. 90-day Moving Average of Sleep Stage in percentage of Total Sleep Time (TST). Moving Averages depict data of the Continuous-2-Year dataset. Blue = Sleep Stage N2; Purple = REM Sleep; Green = Slow Wave Sleep (SWS). Yellow Field = Highlights changes in Sleep Stages occurring possibly at the expense of one another. date format = dd-MMM.

Figure S3 – 90-day Moving-Average (MvA) of Slow Wave Sleep (SWS) and Photoperiod. Black = SWS depicted as the MvA of the Averaged-2-Year dataset ; Red = Photoperiod ^14^; Red dotted horizontal = 12h-photoperiod threshold; Blue dot-dashed (A) = The horizontals indicate the time periods with continuously increasing or decreasing SWS durations; note that the turning points coincide with the time points at which the photoperiod crosses the 12h-threshold (i.e. spring/autumn equinox). Green dashed (B) = The horizontals indicate the time periods with continuously increasing or decreasing photoperiod. date format = dd-MMM.

Figure S4 – 90-day Moving-Average of Sleep Onset Time and End-of-Sleep Time and Statistical Analysis. Blue = average for ALL years (Averaged-2-Year dataset). Red = for the year 2018 (Continuous-2-Year dataset). Yellow = for the year 2019. Below figure: Linear Mixed-Effect Model (LMM) results followed by significant (p < 0.05) post hoc results. Total Sleep Time and REM Sleep were added for reference. date format = MMM-yy.

## Weather differences between years

Over a 30-year period (1989 to 2019), 2018 exhibited the highest median daily mean temperature (12.2°C) ^15^ and daily sunshine duration (5.8 h) ^16^, as well as the lowest median daily relative humidity (68%) ^17^. A direct comparison between 2018 and 2019 reveals that 2018 had a long and cold winter followed by a swift transition to a long summer, as indicated by the mean temperature. Weeks with a median sunshine duration of more than 10 hours occurred more often and were sustained longer in 2018 (*N* = 13 weeks, maximum four consecutive weeks) compared to 2019 (*N* = 8 weeks, maximum two consecutive weeks).

**Figure S5. Overview of environmental parameter for 2018 and 2019. Blue** = 24-h mean temperature in °C (Deutscher Wetterdienst: CDC-Portal ^15^); **Red** = photoperiod in hours (timeanddate.com ^14^) or daily; Yellow = sunshine duration in hours (Deutscher Wetterdienst: CDC-Portal [8]). Black = 30 year average of 24-h mean temperature in °C for each calendar day from 1989 to 2019 (adapted from: Deutscher Wetterdienst: CDC-Portal ^15^). date format = MMM yyyy.

In both years, a distinct high-temperature summer period was observed, involving daily mean temperatures greater than or equal to 27°C (*M_2018≥27°C_* = 26.7°C; *Max_2018≥27°C_* = 38.0°C; *M_2019≥27°C_* = 22.6°C; *Max_2019≥27°C_* = 38.5°C), as well as a low-temperature winter period, involving daily mean temperatures less than or equal to 0°C (*M_2018≤0°C_* = 1.1°C; *Min_2018≤0°C_* = -12.8°C; *M_2019≤0°C_* = 2.9°C; *Min_2019≤0°C_* = -6.9°C) (see Figure 2.A). These periods were shifted forward in 2019 by approximately 1.3 to 1.8 months.

| Discussion Supplementary **Table S5. Estimated Cohort Size for the Linear Mixed-effect Model** | | | | | | | |
| --- | --- | --- | --- | --- | --- | --- | --- |
| **Sleep Parameter** | **SD** (N = 377) | **seasonal window: Mean (N)** | | | | **Effect Size f** | **Total Estimated Cohort Size** |
|  |  | - 91 days | at Min Lag | + 91 days | + 182 days |  |  |
| Total Sleep Time [min] | 69.5 | 418.0(89) | 402.1 ( 98) | 405.0 (73) | 438.4 (117) | 0.22 | 372 |
| Sleep Period Time [min] | 70.4 | 464.8(88) | 450.6 ( 98) | 455.9 (74) | 480.6 (117) | 0.17 | 588 |
| Sleep Latency [min] | 19.8 | 25.5 (89) | 23.5 ( 98) | 25.7 (73) | 25.4 (117) | 0.04 | 8544 |
| REM Latency [min] | 21.0 | 73.5 (86) | 72.0 ( 99) | 59.8 (74) | 65.2 (115) | 0.25 | 288 |
| REM-Sleep [min] | 28.3 | 89.7 (88) | 83.8 (101) | 92.1 (76) | 98.2 (112) | 0.19 | 464 |
| REM-Sleep [% TST] | 5.0 | 21.0 (91) | 20.6 (101) | 22.4 (75) | 22.5 (110) | 0.17 | 604 |
| Slow Wave Sleep [min] | 34.2 | 72.3 (89) | 60.0 (100) | 49.0 (76) | 69.4 (112) | 0.25 | 276 |
| Slow Wave Sleep [% TST] | 8.0 | 17.4 (89) | 15.0 ( 99) | 11.9 (76) | 15.9 (113) | 0.23 | 320 |
| **Abbreviations:** N = number of patients per group; SD = standard deviation **Notes:** Required cohort sizes were calculated using the mean and standard deviation for the “seasonal windows” of the Linear mixed-effect model with α=0.05 and power=0.95 (G*Power: Franz Faul, Universität Kiel, Germany). | | | | | | | |

**References**

1. Dement W, Kleitman N. Cyclic variations in EEG during sleep and their relation to eye movements, body motility, and dreaming. *EEG Clin Neurophysiol*. 1957;9(4):673-690. doi:10.1016/0013-4694(57)90088-3

2. Shrivastava D, Jung S, Saadat M, Sirohi R, Crewson K. How to interpret the results of a sleep study. *J Community Hosp Intern*. 2014;4(5):24983. doi:10.3402/jchimp.v4.24983

3. Ruoff C, Rye D. The ICSD-3 and DSM-5 guidelines for diagnosing narcolepsy: clinical relevance and practicality. *Curr Med Res Opin*. 2016;32(10):1611-1622. doi:10.1080/03007995.2016.1208643

4. Buysse DJ, Reynolds CF, Monk TH, Berman SR, Kupfer DJ. The Pittsburgh sleep quality index: A new instrument for psychiatric practice and research. *Psychiatry Research*. 1989;28(2):193-213. doi:10.1016/0165-1781(89)90047-4

5. Johns MW. A New Method for Measuring Daytime Sleepiness: The Epworth Sleepiness Scale. *Sleep*. 1991;14(6):540-545. doi:10.1093/sleep/14.6.540

6. Krupp LB. The Fatigue Severity Scale: Application to Patients With Multiple Sclerosis and Systemic Lupus Erythematosus. *Arch Neurol*. 1989;46(10):1121. doi:10.1001/archneur.1989.00520460115022

7. Roenneberg T, Wirz-Justice A, Merrow M. Life between Clocks: Daily Temporal Patterns of Human Chronotypes. *J Biol Rhythms*. 2003;18(1):80-90. doi:10.1177/0748730402239679

8. Horne JA, Ostberg O. A self-assessment questionnaire to determine morningness-eveningness in human circadian rhythms. *Int J Chronobiol*. 1976;4(2):97-110.

9. Griefahn B, Kunemund C, Brode P, Mehnert P. Zur Validitat der deutschen Ubersetzung des Morningness-Eveningness-Questionnaires von Horne und Ostberg. The Validity of a German Version of the Morningness-Eveningness-Questionnaire Developed by Horne and Ostberg. *Somnologie*. 2001;5(2):71-80. doi:10.1046/j.1439-054X.2001.01149.x

10. Rosenthal NE, Sack DA, Gillin JC, et al. Seasonal Affective Disorder: A description of the syndrome and preliminary findings with light therapy. *Arch Gen Psychiatry*. 1984;41(1):72-80. doi:10.1001/archpsyc.1984.01790120076010

11. Obradovich N, Migliorini R, Mednick SC, Fowler JH. Nighttime temperature and human sleep loss in a changing climate. *Sci Adv*. 2017;3(5):e1601555. doi:10.1126/sciadv.1601555

12. Anderson JL, Rosen LN, Mendelson WB, et al. Sleep in fall/winter seasonal affective disorder: Effects of light and changing seasons. *Journal of Psychosomatic Research*. 1994;38(4):323-337. doi:10.1016/0022-3999(94)90037-X

13. Seidler A, Weihrich KS, Bes F, de Zeeuw J, Kunz D. Seasonality of human sleep: Polysomnographic data of a neuropsychiatric sleep clinic. *Front Neurosci*. 2023;17:1105233. doi:10.3389/fnins.2023.1105233

14. Thorsen S. Sunrise and sunset in Germany. Time and Date. Accessed November 10, 2022. https://www.timeanddate.com/sun/germany/berlin

15. DWD Climate Data Center (CDC). Daily station observations of mean temperature at 2 m above ground in °C for Germany, version v21.3. Accessed July 28, 2023. https://cdc.dwd.de/portal/

16. DWD Climate Data Center (CDC). Daily station observations of sunshine duration in hours for Germany, version v21.3. July 28, 2023. Accessed July 28, 2023. https://cdc.dwd.de/portal/

17. DWD Climate Data Center (CDC). Daily mean of relative humidity at 2 m above ground in % for Germany, version v21.3. Accessed July 28, 2023. https://cdc.dwd.de/portal/
